# Modeling and Representation of Human Hearts for Volumetric Measurement

**DOI:** 10.1155/2012/389463

**Published:** 2011-11-13

**Authors:** Qiu Guan, Wanliang Wang, Guang Wu

**Affiliations:** ^1^College of Computer Science and Technology, Zhejiang University of Technology, Hangzhou 310023, China; ^2^Guangxi Academy of Sciences, 98 Daling Road, Nanning 530007, China; ^3^DreamSciTech Consulting, Shenzhen 518054, China

## Abstract

This paper investigates automatic construction of a three-dimensional heart model from a set of medical images, represents it in a deformable shape, and uses it to perform volumetric measurements. This not only significantly improves its reliability and accuracy but also makes it possible to derive valuable novel information, like various assessment and dynamic volumetric measurements. The method is based on a flexible model trained from hundreds of patient image sets by a genetic algorithm, which takes advantage of complete segmentation of the heart shape to form a geometrical heart model. For an image set of a new patient, an interpretation scheme is used to obtain its shape and evaluate some important parameters. Apart from automatic evaluation of traditional heart functions, some new information of cardiovascular diseases may be recognized from the volumetric analysis.

## 1. Introduction

The research to diagnose and prevent cardiovascular diseases becomes more important than ever. Thanks to the newly developed technologies in medical imaging and computing, automatic evaluation of patient hearts now becomes possible. This is very useful for diagnosis and treatment. If all kind of cardiovascular diseases were cured, human life could be much longer [1]. Modeling and volumetric measurement have the capability to improve the diagnostic value of cardiac images. Ventricular volume and representation make physician to evaluate the consequences of myocardial infarction according to a glance at the anatomy of the ventricles. Besides, Parameters rely on left ventricular volume such as the ejection fraction, which is a kind of measurement of ventricular ability to pump oxygenated blood through the body. Since cardiologists rely on these values as indicators of cardiac malfunction, it is useful for us to calculate the ventricular volumes [2–6]. 

Researchers have attempted much in this issue, such as medical image acquisition, image processing, feature enhancement and extraction, cardiac boundary segmentation, parameter computation, functional formulation, disease assessment, and so forth. A number of methods have also been attempted for carrying out these works. The contributions can be extensively explored in the literature [7–12]. Typically, Yamamuro et al. carried out a project on two-dimensional (2D) image processing [7]. They evaluate accuracy of cardiac functional analysis with multidetector-row computed tomography (CT) and segmental reconstruction algorithm over a range of heart rates. Various functional parameters of the left ventricle are measured, and they are correlated and agreed with those obtained with magnetic resonance imaging (MRI). For the single-photon emission computed tomography, Germano et al. have developed an algorithm to quantitatively measure left ventricular ejection fraction from gated 99mTc-sestamibi myocardial perfusion images [8]. The algorithm operates in the three-dimensional (3D) space and uses gated short-axis image volumes. It segments the ventricle, estimates and displays endocardial and epicardial surfaces for all gating intervals in the cardiac cycle, calculates the relative left ventricular cavity volumes, and derives the global ejection fraction from the end-diastolic and end-systolic volume. Results show that the automatic segmentation and contouring of the ventricle were very successful of the experimental studies. Relevant programs were also developed to provide clinically useful additional information to complement myocardial perfusion studies in hospitals. However, the limitation of these existing works lies in the lack of cardiac shape completeness, correctness or regularity, and fitting reliability. 

This paper is concerned with modeling and analysis of 3D cardiac model (mainly the ventricle) of human heart and fitting the model to other patients' 3D cardiac image volumes to form the patient's subject-specified model. The model building includes semiautomated registration of shapes for all patients in the database, alignment, and decomposition into a flexible model. For a new case, the model is used to segment and fit 3D cardiac images. Finally quantitative functional analysis of the left ventricle is followed, which plays a very important role in the automatic/aided diagnosis of cardiac diseases. The method utilized in this research is a model-based approach. It includes two meanings. Firstly, the heart model is built by statistical analysis of many existing images from the database which are obtained from several hospitals, which creates a general heart model and possible variation. Secondly, the cardiac shape of a new patient is evaluated from the deviation of its shape from normal cases.

The remainder of this paper is organized as follows. In Sections 2 and 3, we introduce the method for model creation and training. Sections 4 and 5 describe issues of model representation and interpretation. Mathematic method of volumetric measurement is given in Section 6. Example experimental results are given in Section 7. Finally, conclusions are drawn in Section 8.

## 2. Model Creation

The key to quantitative analysis of cardiac functions is to recognize the dynamical and deformable cardiac shape from medical images. In recent decades, researchers have contributed many ideas for segmenting, fitting, and modeling of deformable shapes [13, 14]. The most frequently used ones are Deformable Models, Classification, Global Search, Snakes, Level-set, Eigen-faces and Eigen-patches, and so forth. Some of them are based on minimization of an energy function (e.g., Snakes). However, the energy function method searches the object boundary stopping on most rapid changes of gray-level image profiles. Due to the complexity of deformable object in human body where many other organs are located near it, the gray-level gradients sometimes cannot be used to describe the organ anatomical edges actually. Another typical method, the eigen-patches, is also used to model regions where the shape is assumed to be fixed. However, the problem comes when the medical organ is usually not fixed, for example, when the heart is beating. These require a method for not only modeling the object shape but also its shape variation. From our investigation, a method based on a flexible model is adopted to provide principled means to efficiently parameterize a cardiac shape and its variability. The model is initiated from the active shape modeling method described by Cootes et al. [15–17]. It not only satisfies the requirements of cardiac shape modeling and analysis, but also allows dimensionality reduction of the model for reducing the implementation cost.

**Figure 1:**
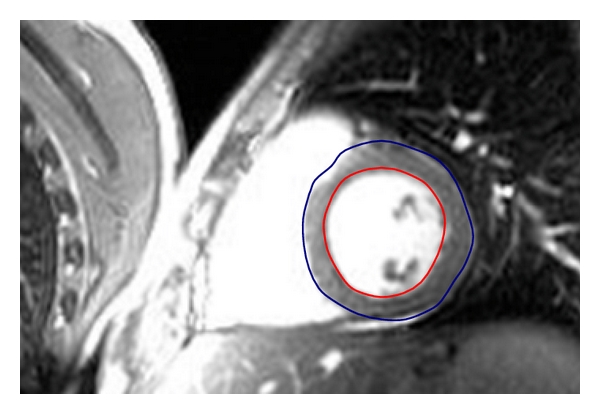
Segmentation of ventricular shapes from its background. In the training step, experienced doctors tell where the accurate boundaries are. The program thus creates a trained model representing the general shape of the hearts.

In the first stage, a statistical model has to be created with a given set of cardiac examples for representing the general shape and its variation. For digital computation, each shape in the training set is represented by a set of labeled feature points, which must be consistent from one shape to another. For instance, a number of 3D points on a ventricle shape should always correspond to the same locations in the biological or anatomical sense.  Figure 1 illustrates a segmentation program for training a general model of the ventricular shape. The segmentation results are also used as the input of the standard for evaluating automatic shape fitting by the proposed model-based approach. 

To build a model of the general shape, it requires labeled training images to represent correspondences among the shape examples. Automatic landmarking methods for this purpose have been thoroughly studied by researchers in recently years. For example, Izard et al. presented a method for landmarking MR images in registering brain structures from different images using a generic algorithm [18]. With a set of such labeled training examples, we need to align them into a common coordinate frame. The Generalized Procrustes Analysis can be used to align training shapes and minimize the sum of squared distances to the mean of the set. In fact, it is to find the transformations *T*_*i*_ which minimize

(1) $$G=\sum {\left|m-T_{i}\left(x_{i}\right)\right|}^{2},$$

where

(2) $$m=\frac{1}{n}\sum T_{i}\left(x_{i}\right),\;\left|m\right|=1,$$

and **x**_*i*_ is a specific shape example in the training set which is represented by a 3*n* element vector (for *n* points of landmarks in 3D space), that is,

(3) $$x={\left(x_{1},y_{1},z_{1},\ldots ,x_{n},y_{n},z_{n}\right)}^{T}.$$

The aligned training set forms a cloud in the 3D space and can be considered as a sample from a probability density function. To reduce the computation cost and memory requirement, we use principal component analysis (PCA) to pick out the main axes of the cloud, and model only the first few, which account for the majority of the variation. For the ventricular model, the first 50 principal eigen vectors are good enough to represent the shape and variation. The general model is then represented as

(4) $$x=\overset{̅}{x}+\Phi b,$$

where $\overset{̅}{x}$ is the mean of the aligned shapes, Φ is a 3*n* × *t*  matrix whose columns are unit vectors along the principal axes, and **b** is a *t* element vector of shape parameters. This means the shape dimension is reduced from 3*n* to *t* by PCA analysis.

This creates a statistical model like the point distribution model, and such a model is used in the flexible model framework to locate new examples in new images. By varying the shape parameters in **b** within limits learnt from the training set, we can generate new plausible shapes. Usually the variance of *b*_*j*_ is *λ*_*j*_ (the eigen value of the *j*th largest in the matrix Φ).

## 3. Model Training by Genetic Algorithm

Genetic algorithm (GA) is introduced as a computational analogy of adaptive systems. It is modeled loosely on the principles of the evolution via natural selection, employing a population of individuals that undergo selection in the presence of variation-inducing operators such as mutation and recombination (crossover). A fitness function is used to evaluate individuals, and reproductive success varies with fitness, and therefore GA is a better way in global search, and passed few years witness its widely applications. There are definitely some laws and sequence of species existing in the nature. Taking medical images, for example, when the artificial factors are excluded, the scale and rotation variety of required images obey the normal distribution. Besides, the information we get from the normal distribution can efficiently help us establish a more reasonable model.

Generally, the statistical model uses the first shape of the training set or an arbitrary shape to be the first meanshape. However, such meanshape is not a “mean” finally. Using GA to form the first meanshape is an alternative choice. We can then get the information of the normal distribution and make the meanshape so as to improve the searching efficiency. Below are some strategies that can be adopted in the fitness function [14, 19].

**Strategy 1 (obtain the model parameters by GA)**

As the amount of GA arithmetic parameters should be controlled when considering the factor of time. The first shape can be used as initial meanshape to form the model, and the model parameters are generated using the GA algorithm. The sum of Euclidean distances between each shape and the meanshape can be taken as the fitness function

(5) $$f_{1}=\sqrt{\overset{n}{\underset{i=1}{\sum }}{\left|\left(\overset{̅}{x}+Pb_{g}\right)-x_{i}\right|}^{2}}.$$

**Strategy 2 (generate the shape directly from GA)**

Directly using the coordinates of the shape points together as the input parameter of GA, the sum of Euclidean distances between each shape and the meanshape can be used in the fitness function. It is an efficient method for the shape without many points. Its computation time will be increased rapidly with the more points

(6) $$f_{2}=\sqrt{\overset{n}{\underset{i=1}{\sum }}\left({\left(x_{g}-x_{i}\right)}^{2}+{\left(y_{g}-y_{i}\right)}^{2}\right).}$$

**Strategy 3 (generate each point one by one)**

Searching the optimum point using GA arithmetic with each point as the input parameter, the fitness of GA is the sum of the distance between the points generated by the GA and the other corresponding points in the training set. Suitable weights are added to reflect the significance of some shapes in the training set. All the points are fitted together as the first mean shape to participate in the align procedure

(7) $$f_{3}=\overset{n}{\underset{i=1}{\sum }}\sqrt{\left({\left(x_{g}-x_{i}\right)}^{2}+{\left(y_{g}-y_{i}\right)}^{2}\right).}$$

GA searches for the optimum solution, and the running time expands more or less when using GA for model generation. Fortunately, this problem is not very serious in the modeling process.

## 4. Model Representation

In this paper, a B-Spline surface model is used to represent the shape and get its volume [20, 21]. By applying matrix form of B-Spline, we obtain a polynomial B-Spline representation with two unitary parameters. Polynomials of B-Spline surfaces make the integral possible. This approach provides an actual volume of B-Spline surface, and it is also convenient and quick. Actually, a part of the B-Spline surface can be represented by the following matrix form:

(8) $$s_{i,j}\left(t,w\right)=T_{k_{1}}M_{i,u}^{k_{1}+1}V_{i,j}^{h}{\left(M_{j,v}^{k_{2}+1}\right)}^{T}W_{k_{2}}^{T},$$

where *T*, *W*, and *M* are basis matrixes in the nonempty intervals for the B-Spline surface. *V* contains the control points:

(9) $$V_{i,j}^{k_{1},k_{2}}=\left[\begin{array}{cccc} V_{i-k_{1},j-k_{2}}^{k_{1},k_{2}} & V_{i-k_{1},j-k_{2}+1}^{k_{1},k_{2}} & \cdots & V_{i-k_{1},j}^{k_{1},k_{2}}\\ V_{i-k_{1}+1,j-k_{2}}^{k_{1},k_{2}} & V_{i-k_{1}+1,j-k_{2}+1}^{k_{1},k_{2}} & \cdots & V_{i-k_{1}+1,j}^{k_{1},k_{2}}\\ \vdots & \vdots & \ddots & \vdots \\ V_{i,j-k_{2}}^{k_{1},k_{2}} & V_{i,j-k_{2}+1}^{k_{1},k_{2}} & \cdots & V_{i,j}^{k_{1},k_{2}} \end{array}\right].$$

Equation (8) can also be rewritten as (10) or split into three scalar equations (11)

(10) $$S_{i,j}\left(t,w\right)=\overset{k_{2}}{\underset{l=0}{\sum }}\;\overset{k_{1}}{\underset{r=0}{\sum }}B_{i,j}\left(r,l\right)t^{r}w^{l},$$

(11) $$\;\begin{array}{c} X\left(t,w\right)=\overset{m}{\underset{i=k_{1}}{\sum }}\overset{n}{\underset{j=k_{2}}{\sum }}\overset{k_{2}}{\underset{l=0}{\sum }}\overset{k_{1}}{\underset{r=0}{\sum }}B_{i,j}^{x}\left(r,l\right)t^{r}w^{l},\\ Y\left(t,w\right)=\overset{m}{\underset{i=k_{1}}{\sum }}\overset{n}{\underset{j=k_{2}}{\sum }}\overset{k_{2}}{\underset{l=0}{\sum }}\overset{k_{1}}{\underset{r=0}{\sum }}B_{i,j}^{y}\left(r,l\right)t^{r}w^{l},\\ Z\left(t,w\right)=\overset{m}{\underset{i=k_{1}}{\sum }}\overset{n}{\underset{j=k_{2}}{\sum }}\overset{k_{2}}{\underset{l=0}{\sum }}\overset{k_{1}}{\underset{r=0}{\sum }}B_{i,j}^{z}\left(r,l\right)t^{r}w^{l}. \end{array}$$

## 5. Interpretation

After we get a statistical model trained from the sets of examples, it is ready to interpret new images. The heart is located in the chest between the lungs behind the sternum and above the diaphragm. It is surrounded by the pericardium. It has the great vessels: the superior and inferior vena cava, the pulmonary artery and vein, and the aorta. The aortic arch lies behind the heart. The esophagus and the spine lie further behind the heart. This knowledge can help the computer to roughly put the cardiac model into a new image volume keeping not too far from the true position for accelerating the interpreting process using the created model.

An iterative method is used for matching the model to images. It iteratively deforms to fit to image volume of the ventricle. The shapes are constrained by statistical derivations to vary only in ways seen in a training set of labeled examples. In addition to the shape model, we require models of the image appearance around each model point. It can be built to represent the statistical variation of the gradient along profiles through the points, normal to the boundary curve at that point. The true boundary position can be found by computing the distance for the statistical profile moving along the image profile [22–25]. Finally the ventricular shape of the patient is fitted by repeating the following two steps until convergence: (1) look along normals through each model point to find the best local match for the model of the image appearance at that point (with minimum distance); (2) update the pose and shape parameters to best fit the model instance to the found points. This is to say that the goal of model fitting is to search best candidate image points near the model and update global transformation, *T*, and parameters, **b**, to minimize

(12) $$\begin{array}{cc} f & ={\left|X-T\left(\overset{̅}{x}+Pb\right)\right|}^{2}\\ & ={\left|X-T\left(\overset{̅}{x}+Pb;X_{c},Y_{c},Z_{c},s,\alpha ,\beta ,\gamma \right)\right|}^{2}, \end{array}$$

where **X** is the temporary model obtained in the immediate steps. This minimization can be achieved by some nonlinear optimizers with iterative approaches. Finally the pose parameters in *T* are fixed, and we get the corresponding shape parameters of the patient.

## 6. Volumetric Measurement

There are mainly three traditional approaches to calculate the ventricular volume. First, the ventricular volume is represented as the volume of a simple shape (e.g., truncated ellipse) or a combination of different figures. This method is simple to perform while the volume is coarse. Secondly, the ventricular volume is regarded as the sum of multiple smaller volumes of similar configuration. Thirdly, the ventricular volume is represented as the volume of B-Spline surface, and the volume is usually computed by using numerical integral such as Simpson's rule and Gauss's rule.

To calculate the volume of B-Spline surfaces, the polynomial expressions of B-Spline surface are given [20]. Let ∂*S*/∂*t* and ∂*S*/∂*w* denote the derivatives of *S*(*u*, *v*) with respect to *t* and *w*, respectively. The volume can be determined by

(13) $$\begin{array}{cc} V & =\overset{m}{\underset{i=k_{1}}{\sum }}\overset{1}{\underset{0}{\int }}A\left(z\left(w\right)\right)z^{\prime }\left(w\right)dw\\ & =\overset{m}{\underset{i=k_{1}}{\sum }}\overset{n}{\underset{j=k_{2}}{\sum }}\iint_{0}^{1}Y\left(t,w\right)\times \frac{\partial X\left(t,w\right)}{\partial t}\times \frac{\partial Z\left(t,w\right)}{\partial w}dt\;dw\\ & =\overset{m}{\underset{i=k_{1}}{\sum }}\overset{n}{\underset{j=k_{2}}{\sum }}\iint_{0}^{1}\overset{\left(3\times k_{2}-1\right)}{\underset{l_{1}=0}{\sum }}\overset{\left(3\times k_{1}-1\right)}{\underset{l_{2}=0}{\sum }}C_{i,j}\left(l_{1},l_{2}\right)\times t^{l_{2}}\times w^{l_{1}}dt\;dw. \end{array}$$

With a *k*_1_ × *k*_2_ order B-Spline surface, it can be finally written as

(14) $$V=\overset{m}{\underset{i=k_{1}}{\sum }}\overset{n}{\underset{j=k_{2}}{\sum }}\left(\overset{\left(3\times k_{2}-1\right)}{\underset{l_{1}=1}{\sum }}\overset{\left(3\times k_{1}-1\right)}{\underset{l_{2}=1}{\sum }}C_{i,j}\left(l_{1},l_{2}\right)\times \frac{1}{l_{2}+1}\times \frac{1}{l_{1}+1}\right),$$

where *C*_*i*,*j*_ is a 3*k*_1_ × 3*k*_2_ matrix which is the product of the three polynomials:

(15) $$\begin{array}{cc} C_{i,j}\left(l_{1},l_{2}\right) & =\overset{2k_{2}-1}{\underset{\begin{array}{c} f=0\\ d=l_{1}-f\\ 0\leq d\leq k_{2} \end{array}}{\sum }}\overset{2k_{1}}{\underset{\begin{array}{c} f_{2}=0\\ d_{2}=l_{2}-f_{2}\\ 0\leq d_{2}\leq k_{1}-1 \end{array}}{\sum }}(\overset{k_{2}}{\underset{\begin{array}{c} r=0\\ s=f-r\\ 0\leq s\leq k_{2}-1 \end{array}}{\sum }}\overset{k_{1}}{\underset{\begin{array}{c} r_{2}=0\\ s_{2}=f_{2}-r_{2}\\ 0\leq s_{2}\leq k_{1} \end{array}}{\sum }}B_{i,j}^{y}\left(r,r_{2}\right)\\ & \;\;\;\;\;\;\;\;\;\;\times s\times B_{i,j}^{x}\left(s,s_{2}\right)\;)\\ & \;\;\;\times d_{2}\times B_{i,j}^{z}\left(d,d_{2}\right). \end{array}$$

## 7. Experiments

**Figure 2:**
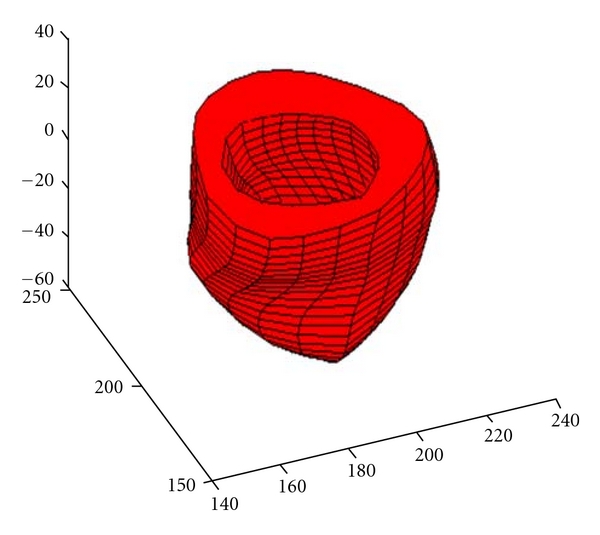
The B-spline surface model of a left ventricle.

Medical images give functional information about the heart while having less information on its anatomy. As it is well known that one of the main diagnostic parameters of interest for physician is its volume. In this paper, some experiments are carried out to construct the left ventricular surfaces fitted by B-spline model (Figure 2). From one cardiac cycle, the changes in volume can be obtained while the heart beats. The ventricular volumes are determined by the algorithms.  Table 1 shows some of the volumes sampled from a cardiac cycle.

## 8. Conclusions

This paper presented a model-based approach for volumetric analysis of human hearts, especially for the ventricles, which is very important for diagnosis and treatment of cardiovascular diseases. The method is based on a flexible model combined with genetic strategies. Based on the flexible model trained from hundreds of patient images, a new patient will be actively analyzed to obtain its individual shape. We also adopted an efficient method for representation of 3D surfaces and provided a corresponding volumetric measurement algorithm of the B-Spline surface. The volumetric algorithm of a B-Spline model is important in working out other functional parameters of human hearts. The pipeline proposed in this paper takes advantage of complete segmentation of the heart shape. It can automatically construct a 3D model from a set of medical images. This not only significantly improves its reliability but also makes it possible to derive valuable information to doctors, such as dynamic volumetric measurements. 

## Figures and Tables

**Table 1:** Volumes at different phases in a cardiac cycle.

Time (ms)	100	300	500	700
Endocardium volume	70.7	49.2	85.2	98.6
Epicardium volume	262	203	294	352
